# Supplementary material for: Transcriptomic plasticity of the hypothalamic osmoregulatory control centre of the Arabian dromedary camel
Source: Commun Biol. 2022 Sep 23;5:1008. doi: 10.1038/s42003-022-03857-0 (PMC9508118; doi:10.1038/s42003-022-03857-0)
Supplement: Supplementary file 2 — Supplementary Information [file 42003_2022_3857_MOESM2_ESM.pdf]

Supplementary tables and figures

Transcriptomic plasticity of the hypothalamic osmoregulatory control centre of the Arabian dromedary camel

Panjiao Lin[1], Benjamin T. Gillard[1], Audrys Pauža[1], Fernando A. Iraizoz[1], Mahmoud A. Ali[2], Andre S. Mecawi[3], Fatma Z. Djazouli Alim[4], Elena V. Romanova[5], Pamela A. Burger[6], Michael P. Greenwood[1†], Abdu Adem[7†#], David Murphy[1†].*

[1]Molecular Neuroendocrinology Research Group, Bristol Medical School: Translational Health Sciences, University of Bristol, Dorothy Hodgkin Building, Bristol, United Kingdom.

[2]Department of Pharmacology, College of Medicine & Health Sciences, United Arab Emirates University, Al-Ain, United Arab Emirates.

[3]Laboratory of Molecular Neuroendocrinology, Department of Biophysics, Paulista School of Medicine, Federal University of São Paulo, São Paulo, Brazil.

[4]University Blida 1, Faculty of Nature and Life Sciences, Department of Biotechnology and Agroecology, Blida, Algeria.

[5]Department of Chemistry and the Beckman Institute, University of Illinois Urbana-Champaign, Urbana, Illinois, United States of America.

[6]Department of Integrative Biology and Evolution, Research Institute of Wildlife Ecology, Vetmeduni Vienna, Vienna, Austria.

[7]Department of Pharmacology, College of Medicine & Health Sciences, Khalifa University, Abu Dhabi, United Arab Emirates.

[†]Equal senior authors

[*]Present address: Gene Therapy and Regulation of Gene Expression Program, Centre for Applied Medical Research – CIMA, University of Navarra, Navarra, Spain

[#]Present address: Department of Pharmacology, Khalifa University, Abu Dhabi, United Arab Emirates

Address correspondence to: David Murphy (d.murphy@bristol.ac.uk); Abdu Adem (abdu.adem@ku.ac.ae).

CT value	mature *AVP*	mature *OXT*	*FOS*	*GAPDH*	*VIP*
**Rostral SON**	16.81	18.63	27.19	21.3	31.11
**Caudal SON**	16.61	18.14	26.68	21.06	33.7

Supplementary Table S1: Preliminary qRT-PCR tests on marker genes for characterizing neuron types in the rostral and caudal SONs of a WD camel.

Raw cycle threshold (CT) values of mature *AVP, OXT, FOS, GAPDH*, and *VIP* in rostral and caudal SONs of a WD camel are recorded. CT values are numbers of cycles required for the fluorescent signal to cross the threshold (background level) and is inversely proportional to the mRNA expression level. Compared to other genes, *VIP*, the suprachiasmatic nucleus (SCN) marker gene, showed barely detectable levels in both rostral and caudal SONs, confirming that there was no SCN contamination in the harvested SON sample.

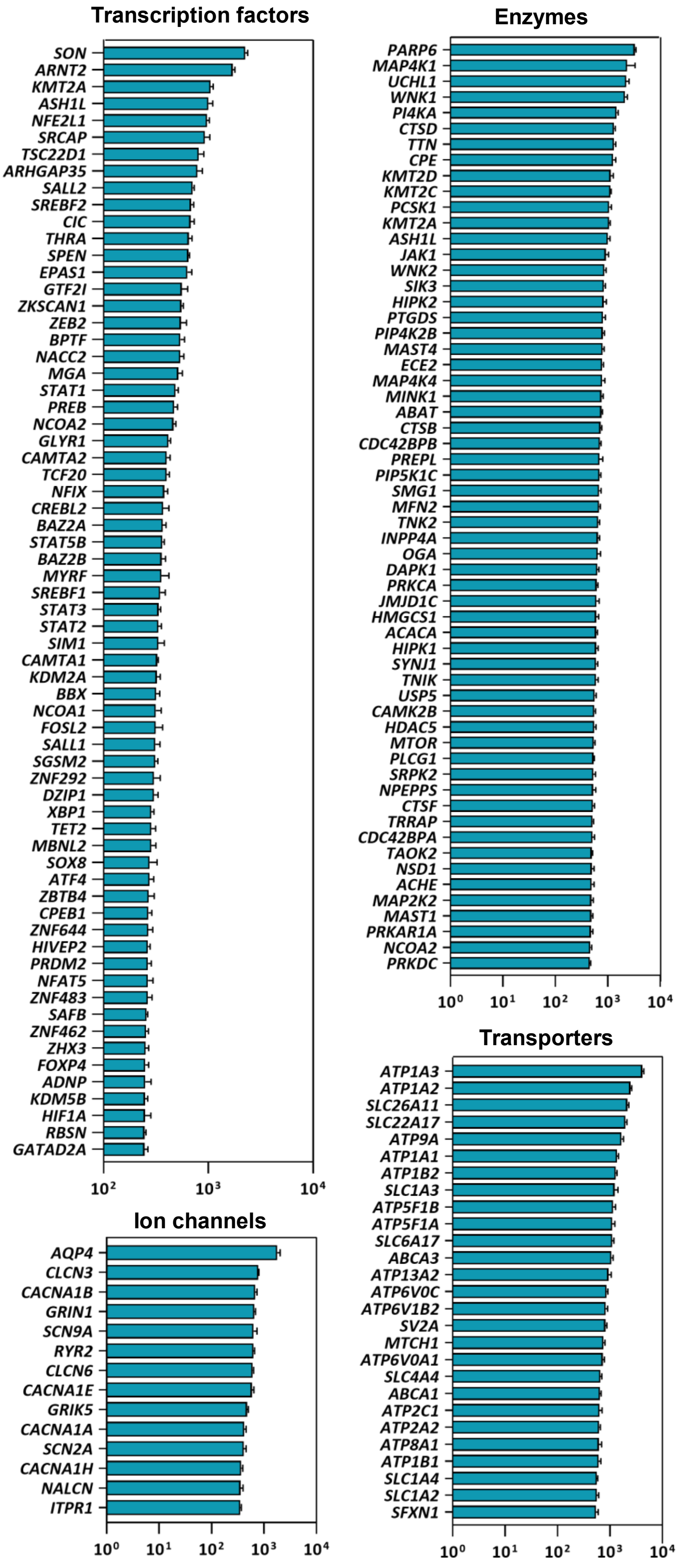

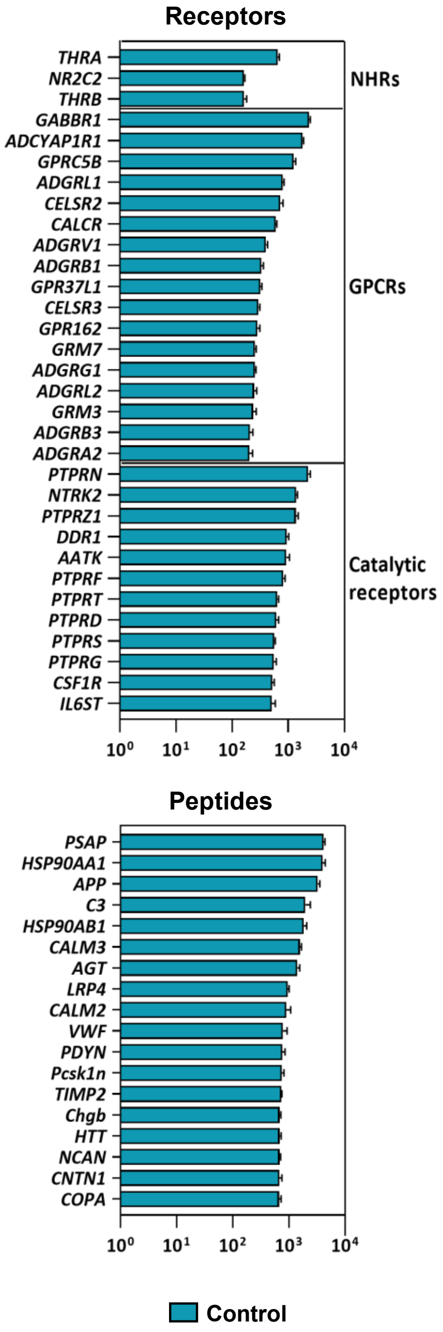

Classification	Number of genes
Transcription factors	66
Enzymes	59
Receptors	32
Peptides	18
Transporters	27
Ion channels	14
Other targets	10

Supplementary Fig. S1: Functional classification of basal (control) genes in camel rostral SON.

Genes were classified according to the functions of their encoded products. From the top 5% most highly expressed genes under control condition, those fit into classifications were categorized into transcription factors, enzymes, receptors (subdivided into NHR: nuclear hormone receptors, GPCR: G protein coupled receptors and catalytic receptors), peptides, transporters, ion channels, and other pharmacological targets (some genes have multiple functions). Data are presented as averaged normalized read counts across all control samples (n=5) ± SEM (standard error of the mean).

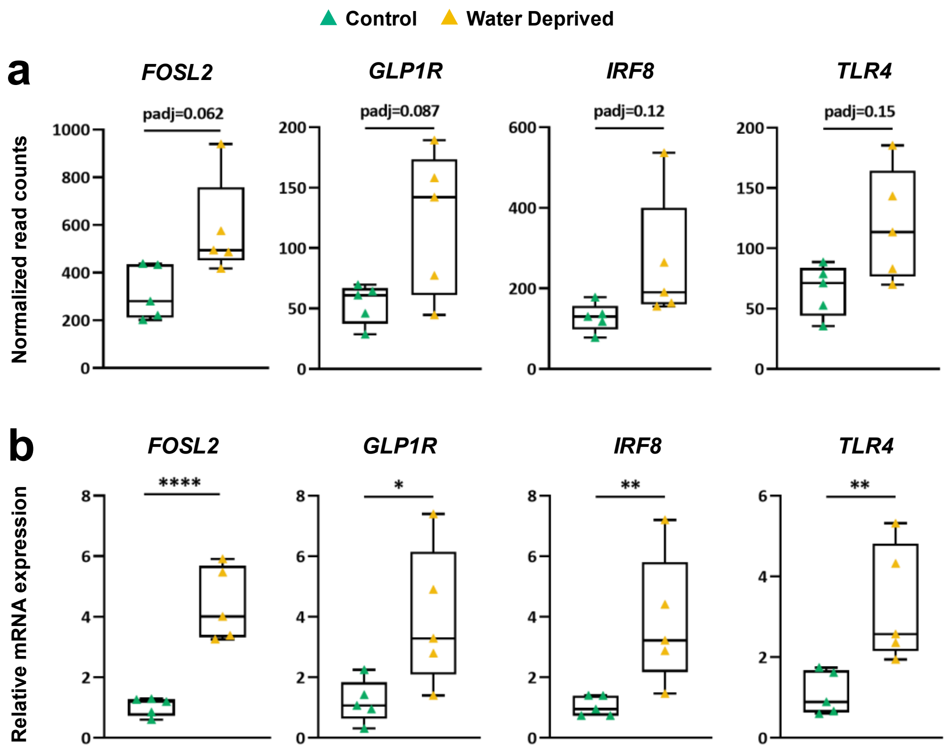

Supplementary Fig. S2: qRT-PCR validation on genes without statistical significance in RNAseq analysis.

Four genes (*FOSL2*, *GLP1R, IRF8, TLR4*) with padj>0.05 (by differential expression analysis between the control and WD camel rostral SON transcriptomes) were tested by qRT-PCR. Results (listed in alphabetical order) are illustrated by box and whisker plots with each individual value shown as a superimposed triangular dot (control: green; WD: yellow). Whiskers represent the minimum and maximum values within a group. The median is shown as a line in the center of the box. **a** Transcript expression level of each gene detected by RNAseq. RNAseq data was analysed by using DESeq2 (Wald test with Benjamin-Hochberg adjustment). padj were labeled as numeric values. **b** Relative mRNA expression levels of each gene detected by qRT-PCR. qRT-PCR data was analysed by using two-way, unpaired t test with Welch correction. *$p \le 0.05$, **$p \le 0.01$, ***$p \le 0.001$, ****$p \le 0.0001$.

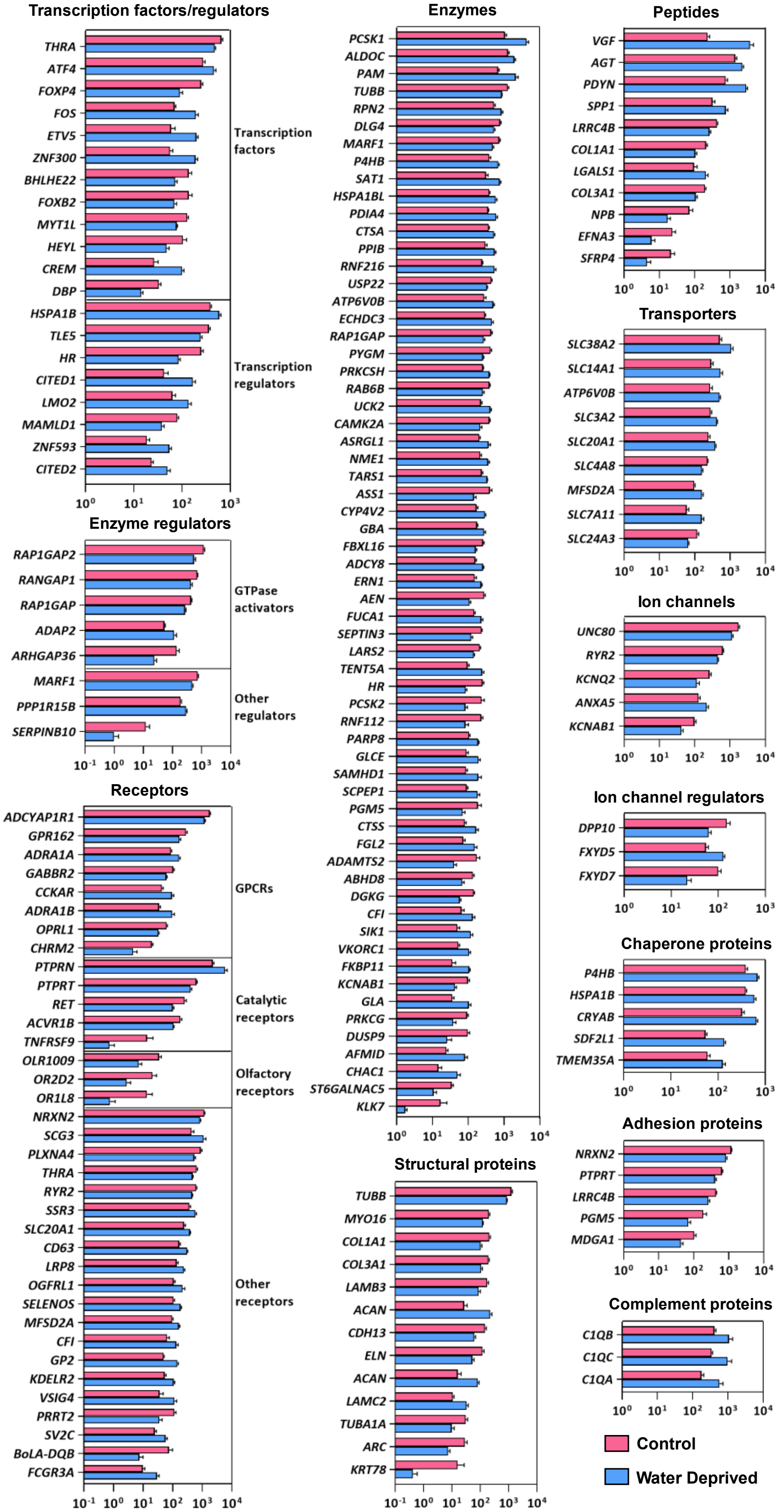

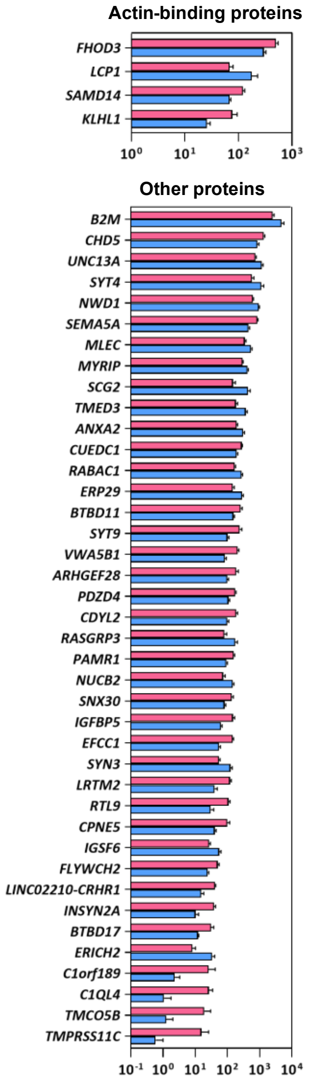

Classification	Number of DEGs
Transcription factors/regulators	20
Enzymes	62
Enzyme regulators	8
Receptors	36
Structural proteins	13
Peptides	11
Transporters	9
Ion channels	5
Ion channel regulators	3
Chaperone proteins	5
Adhesion proteins	5
Complement proteins	3
Actin-binding proteins	4
Other proteins	40

Supplementary Fig. S3: Functional classification of differentially expressed genes by WD in camel rostral SON.

Genes were classified according to the functions of their encoded products. All camel DEGs by WD were functionally classified into transcription factors/regulators, enzymes, enzyme regulators (subdivided into GTPase activators and other regulators), receptors (subdivided into GPCRs: G protein coupled receptors, catalytic receptors, olfactory receptors, and other receptors), structural proteins, peptides, transporters, ion channels, ion channel regulators, chaperone proteins, adhesion proteins, complement proteins, actin-binding proteins and other proteins (some genes have multiple functions). Data are presented as averaged normalized read counts of control or WD samples (*n*=5 for each condition) ± SEM (standard error of the mean).

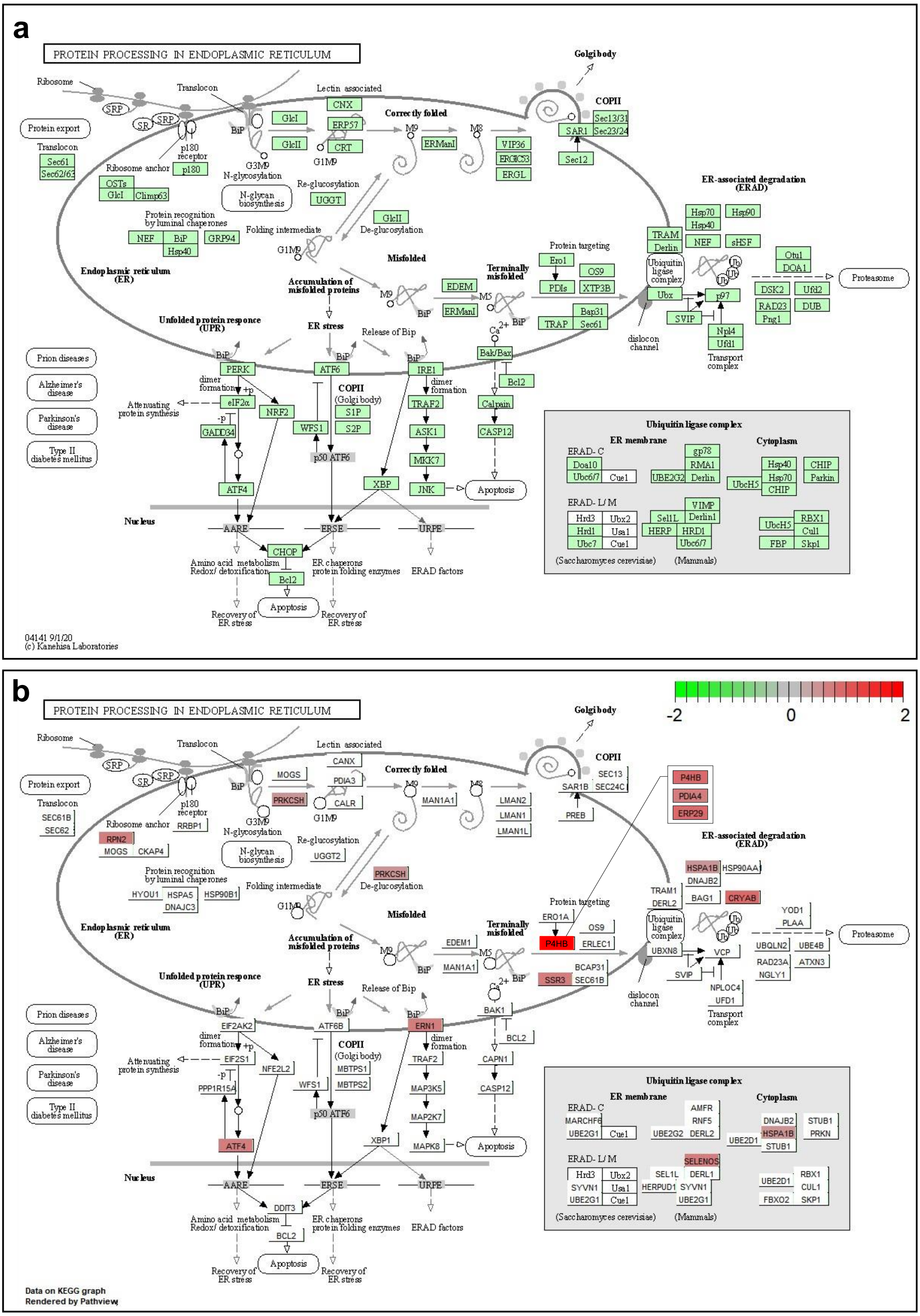

Supplementary Fig. S4: **Over-represented KEGG pathway: protein processing in endoplasmic reticulum.**

a The original KEGG pathway graph. Genes or compounds related to the pathways are visualized as nodes. **b** Pathway rendered with the associated differentially expressed genes (DEGs) regulated by WD in camel rostral SON. Color key: $\log_2$ Fold change (LFC) of gene transcription by WD. Upregulated DEGs (LFC>0) are colored red in the nodes. Downregulated DEGs (LFC<0) are colored green in the nodes (if applicable). The multiple-gene node is expanded into single-gene nodes in the zoom-in window. The products encoded by *PDIA4* and *P4HB* genes are members of the protein disulfide isomerase (PDI) family, the product encoded by *ERP29* forms a chaperone complex together with other components including PDI and BiP[1]. The graphs were generated by using Pathview package (version 1.30.1)[2] in R.

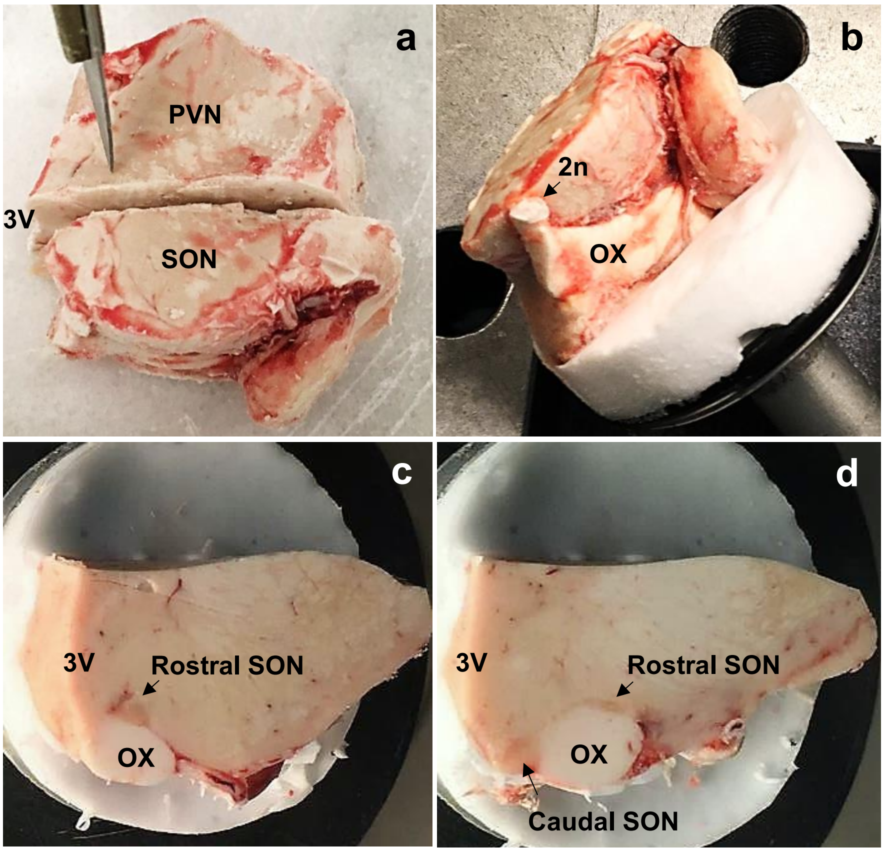

Supplementary Fig. S5: **Locating SON and other neuroanatomic sites in the camel hypothalamus sample.**

The hypothalamus sample was collected from a WD camel. **a** Hypothalamus sample was split into a SON-containing chunk and a PVN-containing chunk. **b** Mounting of the SON-containing chunk to the sample holder. **c** Rostral SON (in coronal plane). **d** Rostral SON and caudal SON (in coronal plane). SON: supraoptic nucleus, PVN: paraventricular nucleus, 3V: third ventricle, 2n: optic nerve, OX: optic chiasm.

Supplementary references

1. Wang, Z., Zhang, H., & Cheng, Q. PDIA4: The basic characteristics, functions and its potential connection with cancer. *Biomed. Pharmacother.* **122**, 109688 (2020).

2. Luo, W., & Brouwer, C. Pathview: an R/Bioconductor package for pathway-based data integration and visualization. *Bioinformatics.* **29**, 1830-1831 (2013).
