# Supplementary material for: Transcriptomic plasticity of the hypothalamic osmoregulatory control centre of the Arabian dromedary camel
Source: Commun Biol. 2022 Sep 23;5:1008. doi: 10.1038/s42003-022-03857-0 (PMC9508118; doi:10.1038/s42003-022-03857-0)
Supplement: Supplementary file 3 — Description of Additional Supplementary Files [file 42003_2022_3857_MOESM3_ESM.pdf]

Description of Additional Supplementary Files

File name: Supplementary Movie S1
Description: The source data behind the 3D mapping of the dromedary camel SON in this paper. This 3D reconstruction was built using 12 RNAscope images (interpolated to 96 planes) of a partial hypothalamus containing SON (in coronal sections). The locations of *AVP* (red) *and OXT* (green) mRNA in the SON of a WD camel indicate the organization of SON. Cellular nuclei stained by DAPI (blue) marks the morphology of tissue structures (especially the optic chiasm as a landmark) were shown at the beginning of the video, then diminished gradually to facilitate the visualization of the SON.

File name: Supplementary Data S1
Description: The source data behind the quality control of cDNA library preparation for RNAseq in this paper.

File name: Supplementary Data S2
Description: The source data behind the figures related to RNAseq differential expression analysis in this paper. Camel and rat RNAseq data were analyzed by using DESeq2 (Wald test with Benjamin-Hochberg adjustment). **a** Original differential expression analysis between WD and control camels without any filtering (22444 genes in total). **b** A subset of (**a**) showing that 21579 genes are detectable for expression in basal (control) state (average normalized read counts across all control samples>0). **c** A subset of (**a**) showing that 21986 genes are detectable for expression regarding collectively to both control and WD groups (baseMean>0). **d** Original differential expression analysis between WD and control rats without any filtering (32402 genes in total). Ensembl was used for retrieving rat gene annotations using AnnotationDbi (version 1.50.3) packages. A total of 2247 DEGs (Benjamini-Hochberg correction, padj≤0.05) were identified. CON: control. WD: water deprived. BaseMean: the average of the normalized count values, dividing by size factors, taken over all samples. log2FoldChange: the effect size estimate. LFC value indicates how much the gene or transcript's expression have changed between the comparison and control groups. LFC value is reported on a logarithmic scale to base 2. lfcSE: the standard error estimate for the LFC estimate. Stat: the value of the test statistic for the gene or transcript. Pvalue: P-value of the test for the gene or transcript. padj: adjusted P-value for multiple comparison for the gene or transcript. **e** Directions of change in expression of the common DEGs between WD camel and rat. UP: upregulation in WD compared to control. DOWN: downregulation in WD compared to control. **f** Simple linear regression and spearman correlation tests on the LFC values of the common DEGs between camel and rat. **g** Simple linear regression and spearman correlation tests on absolute LFC and -log10padj values of the common DEGs in camel or rat. **h** Wilcoxon matched-pairs signed rank test (two-tailed) for comparing the absolute LFC values of the common DEGs between camel and rat.

File name: Supplementary Data S3
Description: The source data behind the figures related to the functional classifications of genes in this paper. **a-i** Functional classifications of the camel basal (control) genes. **a** Transcription factors. **b** Peptides. **c** Catalytic receptors. **d** Enzymes. **e** G protein-coupled receptors (GPCRs). **f** Nuclear hormone receptors (NHRs). **g** Ion channels. **h** Transporters. **i** Other pharmacological targets. **j** Functional classifications of the camel DEGs.

File name: Supplementary Data S4
Description: The source data behind the figures related to the Gene Ontology analysis in this paper.

Over-representation analysis were performed based on a set of genes (with all genes expressed in camel or rat SON transcriptome as background) referring to the human (for camel) or rat (for rat) biological process and KEGG pathway databases. Benjamin-Hochberg (BH) adjustment was applied to reduce the false discovery rate (FDR). **a** Gene ontology on all camel DEGs. **b** Gene ontology on common DEGs between camel and rat. **c** Gene ontology on DEGs unique to camel. GeneRatio=k/n (k is size of the overlap of input genes with the specific geneset, n is size of the overlap of input genes with all the members of the collection of genesets); BgRatio=M/N (M is size of the geneset, N is the total number of genes in the background distribution); pvalue: calculated using hypergeometric distribution; p.adjust: pvalue corrected by BH method; qvalue: pvalue adjusted to calculate FDR for multiple testing; count: size of the overlap of input genes with the specific geneset.

File name: Supplementary Data S5
Description: The source data of the sequences of RNAscope probes, PCR and qRT-PCR primers in this paper. **a** PCR primers designed for the cloning and sequencing of the dromedary *AVP* and *OXT* genes (genomic DNA). **b** RNAscope probes of the dromedary *AVP* and *OXT* mRNAs were designed based on the exonic sequences of the two genes. **c** qRT-PCR primers designed for dromedary genes. *hnAVP*: heteronuclear *AVP*, *hnOXT*: heteronuclear *OXT.* For genes with multiple transcript variants, primers were designed based on the common sequence across all transcript variants.

File name: Supplementary Data S6
Description: The source data behind the statistical analysis of the plasma angiotensin II measurements and qRT-PCR tests in this paper. **a** For plasma ANG II measures over the 20 days of WD in comparison to control, data was analysed using two-way repeated measures ANOVA with Šídák's multiple comparisons test. **b** For plasma ANG II of the rehydrated group over 72 hrs of rehydration in comparison to control and WD states, data was analysed using one-way mixed-effects model (restricted maximum likelihood) for repeated measures with Tukey's multiple comparison test. For the mature and heteronuclear *AVP* and *OXT* genes and DEGs identified by RNAseq (*AGT, ATF4, ATP6V0B, C1QB, CAMK2A, CCKAR, COL1A1, COL3A1, CREM, CTSA, FOS, GABBR2, PCSK1, PDYN, PTPRN, SCG2, VGF, ERN1, P4HB, SELENOS* and *SSR3*), qRT-PCR data was analysed by using Brown-Forsythe and Welch one-way ANOVA with Dunnett T3 post-hoc test. For *FOSL2, GLP2R, IRF8* and *TLR4* genes, qRT-PCR data was analysed using two-way, unpaired t test with Welch correction.
