# Supplementary material for: Transcriptomic plasticity of the hypothalamic osmoregulatory control centre of the Arabian dromedary camel
Source: Commun Biol. 2022 Sep 23;5:1008. doi: 10.1038/s42003-022-03857-0 (PMC9508118; doi:10.1038/s42003-022-03857-0)
Supplement: Supplementary file 11 — Reporting Summary [file 42003_2022_3857_MOESM11_ESM.pdf]

nature portfolio

Corresponding author(s): David Murphy, Abdu Adem

Last updated by author(s): Jul 21, 2022

Reporting Summary

Nature Portfolio wishes to improve the reproducibility of the work that we publish. This form provides structure for consistency and transparency in reporting. For further information on Nature Portfolio policies, see our Editorial Policies and the Editorial Policy Checklist.

Statistics

For all statistical analyses, confirm that the following items are present in the figure legend, table legend, main text, or Methods section.

n/a	Confirmed	
☐	☒	The exact sample size (n) for each experimental group/condition, given as a discrete number and unit of measurement
☐	☒	A statement on whether measurements were taken from distinct samples or whether the same sample was measured repeatedly
☐	☒	The statistical test(s) used AND whether they are one- or two-sided<br>*Only common tests should be described solely by name; describe more complex techniques in the Methods section.*
☒	☐	A description of all covariates tested
☐	☒	A description of any assumptions or corrections, such as tests of normality and adjustment for multiple comparisons
☐	☒	A full description of the statistical parameters including central tendency (e.g. means) or other basic estimates (e.g. regression coefficient) AND variation (e.g. standard deviation) or associated estimates of uncertainty (e.g. confidence intervals)
☐	☒	For null hypothesis testing, the test statistic (e.g. F, t, r) with confidence intervals, effect sizes, degrees of freedom and P value noted<br>*Give P values as exact values whenever suitable.*
☒	☐	For Bayesian analysis, information on the choice of priors and Markov chain Monte Carlo settings
☒	☐	For hierarchical and complex designs, identification of the appropriate level for tests and full reporting of outcomes
☐	☒	Estimates of effect sizes (e.g. Cohen's d, Pearson's r), indicating how they were calculated

Our web collection on statistics for biologists contains articles on many of the points above.

Software and code

Policy information about availability of computer code

Data collection	No software or custom code was used for data collection in this paper.
Data analysis	Software/software package: ImageJ (bundled with 64-bit Java 1.8.0_112), MATLAB version 9.4.0 (R2018a), Spliced Transcripts Alignment to a Reference (STAR, version 2.5.3a), R version 4.0.3, FeatureCounts (inbuilt in Rsubread version 2.4.3), DESeq2 version 1.30.0, ggplot2 version 3.3.3, ClusterProfiler version 3.18.1, AnnotationDbi version 1.52.0, Org.Hs.eg.db version 3.12.0, Graphpad Prism version 9.1.0.<br><br>This paper does not report original code. All code was adapted from the user manual of the software packages and is available from the corresponding author upon reasonable request. Any additional information required to reanalyze the data reported in this paper is available from the corresponding author upon reasonable request.

For manuscripts utilizing custom algorithms or software that are central to the research but not yet described in published literature, software must be made available to editors and reviewers. We strongly encourage code deposition in a community repository (e.g. GitHub). See the Nature Portfolio guidelines for submitting code & software for further information.

Data

Policy information about availability of data

All manuscripts must include a data availability statement. This statement should provide the following information, where applicable:

- Accession codes, unique identifiers, or web links for publicly available datasets
- A description of any restrictions on data availability
- For clinical datasets or third party data, please ensure that the statement adheres to our policy

The dromedary camel AVP (accession number: OM963135) and OXT (accession number: OM963134) gene sequences have deposited to GenBank. The data underlying the transcriptomic analyses, including raw FASTQ files, bulk RNAseq counts, DESeq2-normalized data and project metadata, have been deposited in NCBI's Gene Expression Omnibus (GEO) and are publicly available as of the date of publication (accession number: GSE198577). All software packages used to analyse the data are described in the Methods section, and are common, well-established tools used in omics studies.

Human research participants

Policy information about studies involving human research participants and Sex and Gender in Research.

Reporting on sex and gender	This study did not involve human research participants.
Population characteristics	This study did not involve human research participants.
Recruitment	This study did not involve human research participants.
Ethics oversight	This study did not involve human research participants.

Note that full information on the approval of the study protocol must also be provided in the manuscript.

Field-specific reporting

Please select the one below that is the best fit for your research. If you are not sure, read the appropriate sections before making your selection.

☒ Life sciences ☐ Behavioural & social sciences ☐ Ecological, evolutionary & environmental sciences

For a reference copy of the document with all sections, see nature.com/documents/nr-reporting-summary-flat.pdf

Life sciences study design

All studies must disclose on these points even when the disclosure is negative.

Sample size	This animal experiment was an international collaboration by different institutions. Based on the study design by all collaborators, the sample size was confirmed to be: control group (n=5), water deprived group (n=8), rehydrated group (n=6). The transcriptomic analysis in our study is benefited by the sample size no less than 5 to obtain sufficient statistical power.
Data exclusions	Three samples that did not pass the cDNA library quality control were excluded for RNAseq. Details are available in Methods section.
Replication	For key genes that were identified to be changed in expression by water deprivation in RNAseq, we performed qRT-PCR as a validation, with the majority of the tested genes successfully validated. The study was not replicated on a separate group of dromedary camels because they are not easily accessed as non-model animals.
Randomization	The organisms were randomly allocated into experimental groups.
Blinding	Group allocations were only disclosed to the workers in the rance house who maintained the animals, hence the investigators were blinded during sampling and data collection. The investigators who perfomred the RNAseq were also blinded from the group allocation.

Reporting for specific materials, systems and methods

We require information from authors about some types of materials, experimental systems and methods used in many studies. Here, indicate whether each material, system or method listed is relevant to your study. If you are not sure if a list item applies to your research, read the appropriate section before selecting a response.

Materials & experimental systems

n/a	Involved in the study
☐	☒ Antibodies
☒	☐ Eukaryotic cell lines
☒	☐ Palaeontology and archaeology
☐	☒ Animals and other organisms
☒	☐ Clinical data
☒	☐ Dual use research of concern

Methods

n/a	Involved in the study
☒	☐ ChIP-seq
☒	☐ Flow cytometry
☒	☐ MRI-based neuroimaging

Antibodies

Antibodies used

Rabbit anti-angiotensin II antibody (Peninsula Laboratories Inc.; Cat#T-4007.0050)

Validation

Plasma ANG II concentration was determined by specific radioimmunoassay using T-4007 antibody from Peninsula Laboratories, Inc. (San Carlos, CA, USA) as described by Mecawi et al. (2013).

Mecawi, A.S., Vilhena-Franco, T., Fonseca, F.V., Reis, L.C., Elias, L.L., and Antunes-Rodrigues, J. (2013). The role of angiotensin II on sodium appetite after a low-sodium diet. J. Neuroendocrinol. 25, 281-291.

Animals and other research organisms

Policy information about studies involving animals; ARRIVE guidelines recommended for reporting animal research, and Sex and Gender in Research

Laboratory animals

This study did not involve laboratory animals.

Wild animals

This study did not involve wild animals.

Reporting on sex

The findings in this study apply to only males. This study was designed to involve only male dromedary camels to eliminate any sex bias.

Field-collected samples

The camels were supplied with alfalfa hay as feed and were ranch-housed outside Al Ain, United Arab Emirates during the hot months (April and May) of 2016, under careful veterinary supervision. After a short adaptive phase, the camels were divided into a control group (n=5), a WD group (n=8), and a rehydrated group (n=6). The control group had free access to food and water throughout the experimental period. The WD group was supplied with food ad libitum but without access to water for 20 days. The rehydrated group was WD for 20 days followed by an unlimited water supply for 3 days. After the experiment, the camels were sacrificed in the local central abattoir for human consumption.

Ethics oversight

This study was approved by the Animal Ethics Committee of the United Arab Emirates University (approval ID: AE/15/38) and the University of Bristol Animal Welfare and Ethical Review Board.

Note that full information on the approval of the study protocol must also be provided in the manuscript.
